# P-398. Tiny Patients, Big Questions: Assessing Cefazolin Dosing in Pediatrics

**DOI:** 10.1093/ofid/ofaf695.615

**Published:** 2026-01-11

**Authors:** Michaela Mahnke, Jennifer A Schweiger, Lee Morris, Amanda Lefemine

**Affiliations:** Advocate Health: Atrium Health, Charlotte, North Carolina; Advocate Health: Atrium Health Antimicrobial Support Network, Charlotte, North Carolina; Advocate Health: Atrium Health Antimicrobial Support Network, Charlotte, North Carolina; Advocate Health: Atrium Health Antimicrobial Support Network, Charlotte, North Carolina

## Abstract

**Background:**

Cefazolin is a widely used and generally well-tolerated antibiotic for pediatric patients. Due to limited clinical evidence available in this population, dosing practices vary significantly, and the incidence of adverse effects remains uncertain.

**Table 1.**
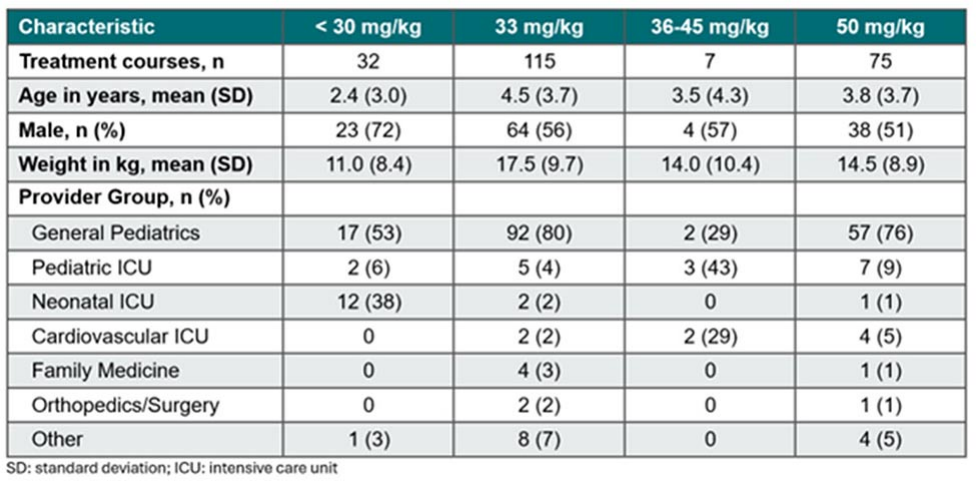
Baseline Characteristics

**Figure 1.**
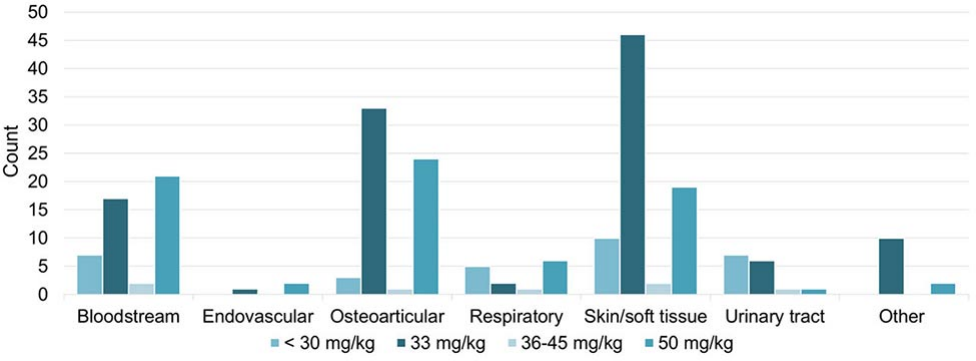
Cefazolin Dosing by Indication

**Methods:**

This retrospective study included hospitalized pediatric patients at Levine Children’s Hospital in Charlotte, NC that received at least 6 sequential doses of cefazolin from August 2023 through July 2024. Neonates, patients ≥ 40 kg, and orders with an indication of surgical prophylaxis were excluded. Additional laboratory data was reviewed for a safety analysis if the patient received ≥ 21 doses. Patients were evaluated in groups based on their dose and weight: < 30 mg/kg, 33 mg/kg, 36-45 mg/kg, and 50 mg/kg. The primary objective was to characterize cefazolin prescribing practices with a focus on dosing strategy by indication. Secondary objectives included evaluating the impact of different dosing regimens on incidences of adverse effects.

**Figure 2.**
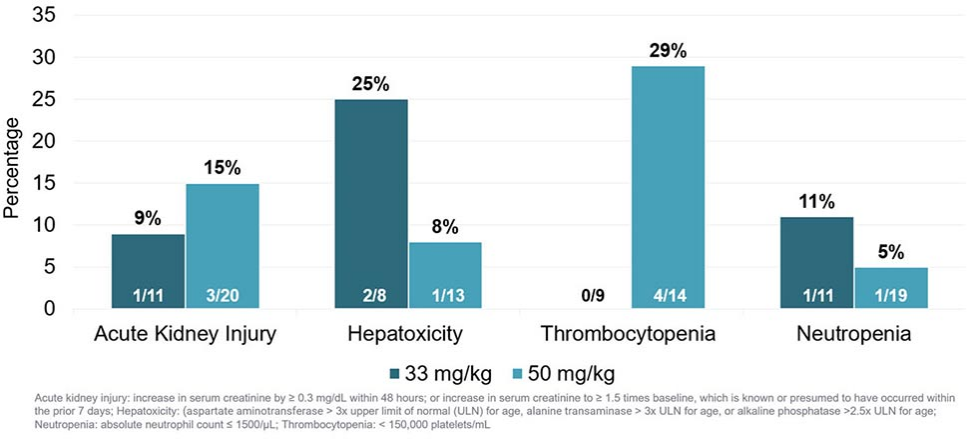
Incidence of Adverse Effects

**Results:**

Of 229 patient encounters included, 32 received cefazolin doses < 30 mg/kg, 115 received 33 mg/kg, 7 received 36-45 mg/kg, and 75 received 50 mg/kg. The mean patient age was 4 years, and most orders were placed by the general pediatrics team (Table 1). The most common indications were skin/soft tissue (n=77), osteoarticular (n=61), and bloodstream infections (n=47). Patients with skin/soft tissue infections were prescribed cefazolin 33 mg/kg a majority of the time. The initial doses for bloodstream and osteoarticular infections varied (Figure 1). Adjustments in dosing strategy occurred during 11% (25/229) of courses. Among the 58 courses reviewed in the safety analysis, incidence of adverse effects was low (Figure 2).

**Conclusion:**

Wide variation in cefazolin dosing practices was observed, but the incidence of adverse effects was low, with minimal differences noted among the various dosing strategies. The potential benefits of optimizing drug exposure may outweigh the risks of higher dosing, particularly in severe infections. Future studies assessing the effect of dosing strategies on clinical outcomes are warranted.

**Disclosures:**

All Authors: No reported disclosures

